# Size effect of parallel-joint spacing on uniaxial compressive strength of rock

**DOI:** 10.1371/journal.pone.0257245

**Published:** 2021-09-20

**Authors:** Gaojian Hu, Gang Ma

**Affiliations:** 1 School of Civil Engineering, Shaoxing University, Shaoxing, Zhejiang, China; 2 Key Laboratory of Rock Mechanics and Geohazards of Zhejiang Province, Shaoxing, Zhejiang, China; 3 Zhejiang Collaborative Innovation Center for Prevention and Control of Mountain Geologic Hazards, Shaoxing, Zhejiang, China; China University of Mining and Technology, CHINA

## Abstract

The existence of parallel joints has an impact on the size effect of the uniaxial compressive strength of rock, but the relationship is yet to be obtained. In this paper, the influence of parallel-joint spacing on the size effect and characteristic size of rock uniaxial compressive strength is studied by establishing five types of parallel-joint-spacing simulation schemes. The influence of parallel-joint spacing on the size effect of rock uniaxial compressive strength is explored by analyzing the stress–strain curves of rocks with different parallel-joint spacings and rock sizes. The relationship between the uniaxial compressive strength and the size of the rock with parallel joints and its special mathematical model are obtained, and the particular form of the compressive-strength characteristic size and parallel-joint spacing is obtained.

## Introduction

Compressive strength is one of the mechanical properties of rock strength and has a size effect (SE). The existence of parallel joints (PJs) changes the compressive strength of rocks, and in-depth study of the SE of the compressive strength of PJed rocks has important theoretical and practical significance for the safe construction and stability analysis of rock slopes, tunnels, and other rock-mass projects.

Scholars have studied the SE of the compressive strength of rocks. Fu [1] obtained the law governing the SE of rock compressive strength by preparing hard rock specimens with different height-to-diameter ratios for uniaxial compression tests. Lu et al. [2] conducted uniaxial compression tests on soft rocks with different height-to-diameter ratios based on gray relational analysis to study the SE of soft rock strength. Meng et al. [3] studied the SE of the strength of mudstone and sandstone based on numerical simulations. The above scholars studied the strength of different types of rocks and found that the compressive strength of rocks has an SE. With the deepening of research, scholars have also analyzed the reasons for the SE of rock strength. Yan et al. [4] used the FLAC3D software to study the SE of heterogeneous rock strength and showed that the SE of rock strength is related to the heterogeneity of the rock.

Scholars have used numerical simulations to study the SE of rock strength. Ma et al. [5] studied the SE of the strength of jointed rock masses by using the 3DEC software based on the Monte Carlo method. Chen et al. [6] used the PFC (Particle Flow Code) software to simulate different scales of discontinuous jointed rocks and obtained the influence of the penetration rate on the rock SE. Luo et al. [7] used the RFPA (Realistic Failure Process Analysis) software to perform uniaxial compression numerical simulations on rock under three different boundary conditions and found that rock strength has an SE. Of the various types of numerical simulation software, the RFPA numerical code developed by Tang [8] performs well in simulating the deformation and failure of rock materials. For example, through comparative analysis of laboratory tests and RFPA3D, Wang et al. [9] showed that RFPA was consistent with the laboratory tests in studying the SE of rock strength. The above research shows that numerical simulations have certain advantages in studying the SE of rock strength.

Scholars have also studied the SE of rock compressive strength by establishing formulas. Liu et al. [10] proposed an empirical formula to describe the effect of rock size based on laboratory tests, and it was verified in seven types of rock uniaxial compression tests. Li et al. [11] obtained the relationship between the compressive strength and particle size of rockfill particles based on the discrete model. However, those studies rarely considered the influence of the PJ spacing (PJS).

The quantitative description of the SE of rock strength is the characteristic size or representative elementary volume (REV) [12]. Based on case analysis, Gasmi et al. [13] proposed two representative characteristic sizes, i.e., the geometric REV and the mechanical REV. Scholars have used different methods to evaluate the characteristic size of rocks. For example, Ying et al. [14] proposed a method for estimating rock-mass REV based on the volumetric fracture strength (*P*_32_) and statistical tests. Loyola et al. [15] evaluated the REV of fractured rock by using the central limit theorem. Scholars have obtained the characteristic size of rocks based on field data, theoretical models, and probability statistics. Chong et al. [16] studied the SE of jointed rock masses and estimated the REV size of the rock mass to be 10 m × 10 m. Liang et al. [17] studied the SE of the uniaxial compressive strength (UCS) of rock mass based on the Monte Carlo method and obtained a characteristic size of 14 m × 14 m. Scholars have also analyzed theoretically the factors that affect the characteristic size. For example, Niazmand et al. [18] studied the influence of different constraints on the REV based on the discrete-fracture-network–discrete-element method, and Chen et al. [19] studied the influence of rock mass (*B*_*z*_) on the REV of rock. The above research obtained the characteristic size of rock from different methods and theories but did not consider the influence of PJS on characteristic size. Few scholars have established mathematical models of characteristic size and PJS.

PJS affects the compressive strength of rock. Yang et al. [20] and Song et al. [21] studied the effect of joint spacing on the peak strength of discontinuous jointed rock under uniaxial compression. Liu et al. [22,23] studied the influence of joint spacing on rock fatigue mechanism and the fatigue mechanical properties of jointed rock models under different cyclic conditions. Liu et al. [24] proposed a damage constitutive model for the strength characteristics of intermittently jointed rocks under uniaxial compression. Bao et al. [25] obtained a positive linear relationship between joint spacing and sandstone thickness based on the study of joint outcrops. However, few scholars have considered the influence of rock size on rock strength in the study of how joint spacing affects the mechanical properties of rock.

Herein, the influences of (i) rock size on rock UCS and (ii) PJS on characteristic size are studied by establishing five numerical simulation schemes. The relationship between the rock UCS and the size of the PJed rock is explored under each numerical simulation scheme by analyzing the stress–strain curves of different PJed rock sizes, and the relationships between (i) the UCS and the size of PJed rock and (ii) the uniaxial compressive characteristic size and the PJS are explored by establishing mathematical models.

## Theoretical basis and simulation scheme

The software used in this simulation is RFPA, which is a numerical calculation method for simulating inhomogeneous materials developed based on finite-element theory and statistical damage theory. This method accounts for the non-uniformity and randomness of materials and combines the statistical distribution assumptions of material properties into the finite-element method. It is assumed that the mechanical properties of discretized meso-primitives obey a certain statistical distribution law, thereby establishing the relationship between the meso-medium and macro-medium mechanical properties. The method is described by the Weibull statistical distribution function, whose formula is

$$\varphi (\alpha )=\frac{m}{\alpha_{0}}\cdot {\left(\frac{\alpha }{\alpha_{0}}\right)}^{m-1}\cdot e^{-{\left(\frac{\alpha }{\alpha_{0}}\right)}^{m}}$$ (1)

where *α* is a basic mechanical parameter of the material medium (e.g., elastic modulus, strength, Poisson’s ratio, weight), *α*_0_ is the average value of *α*, *m* is the property parameter of the distribution function, whose physical meaning reflects the uniformity of the material medium, and *φ*(*α*) is the statistical distribution density of the material elementary mechanical property.

### Strength theory

The failure criterion used in this simulation is the Mohr–Coulomb yield criterion. In RFPA, the principal stresses *σ*_1_, *σ*_2_, and *σ*_3_ are used in the simulation by using this yield criterion, and the inequalities

$$\sigma_{1}\leq \sigma_{2}\leq \sigma_{3}$$ (2)

are obeyed. The corresponding principal strain increments Δ*e*_1_, Δ*e*_2_, and Δ*e*_3_ are decomposed as

$$\Delta e_{i}=\Delta e_{i}^{e}+\Delta e_{i}^{p}\;(i=1,2,3)$$ (3)

where the superscripts *e* and *p* refer to the elastic and plastic parts, respectively.

According to the principal stress and principal strain, the incremental expression of Hooke’s law is

$$\left\{ \begin{array}{l} \Delta \sigma_{1}=\alpha_{1}\Delta e_{1}^{e}+\alpha_{2}(\Delta e_{2}^{e}+\Delta e_{3}^{e})\\ \Delta \sigma_{2}=\alpha_{1}\Delta e_{2}^{e}+\alpha_{2}(\Delta e_{1}^{e}+\Delta e_{3}^{e})\\ \Delta \sigma_{3}=\alpha_{1}\Delta e_{3}^{e}+\alpha_{2}(\Delta e_{1}^{e}+\Delta e_{2}^{e}) \end{array}\right.$$ (4)

where *α*_1_ and *α*_2_ are the material constants defined by the shear elastic modulus *G* and bulk modulus *K* as given by

$$\alpha_{1}=K+4G/3,\;\alpha_{2}=K-2G/3$$ (5)

**Fig 1:**
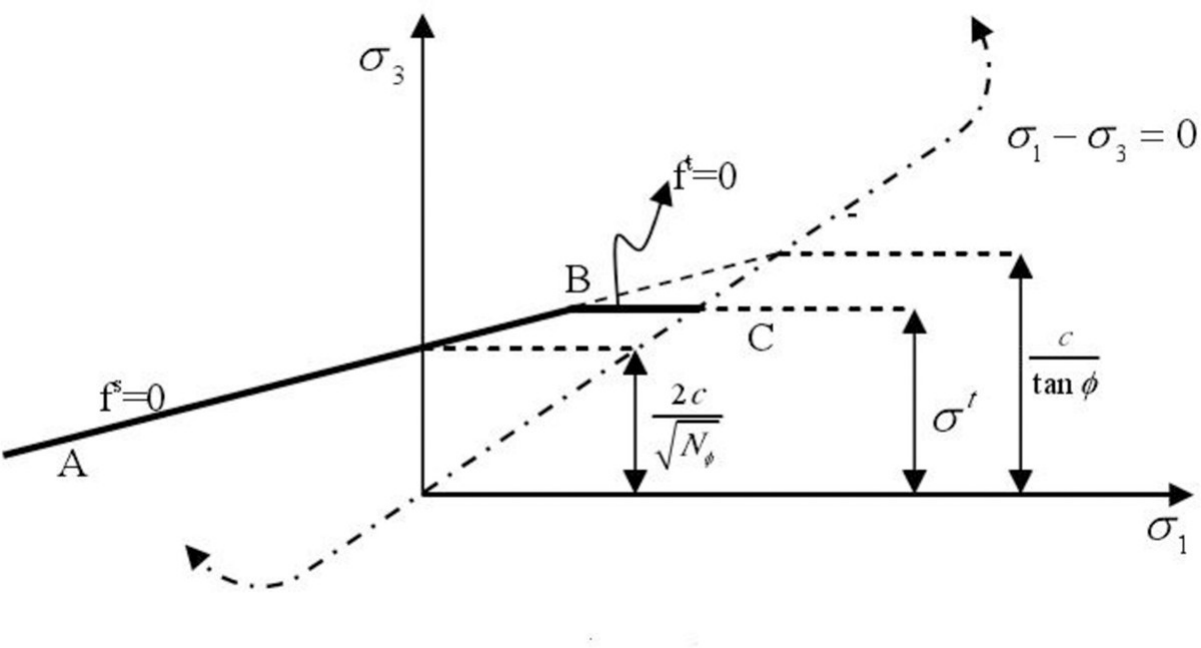
Mohr–Coulomb model failure criteria.

According to the Eqs 2–5, the failure criterion is described in the plane (*σ*_1_, *σ*_2_), as shown in Fig 1. From the Mohr–Coulomb yield function, the damage envelope from point A to point B can be obtained as

$$f^{s}=\sigma_{1}-\sigma_{3}N_{\varphi }-2c\sqrt{N_{\varphi }}$$ (6)

The pull failure function from point B to point C is

$$f^{t}=\sigma_{t}-\sigma_{3}$$ (7)

$$N_{\varphi }=\frac{1+\sin\varphi }{1-\sin\varphi }$$ (8)

where *φ* is the internal friction angle, *c* is the cohesion, and σ_t_ is the tensile strength.

### Simulation scheme and model establishment

The focus herein is on how the size of rock with PJs influences the UCS. Five sets of numerical simulation schemes were established with a PJS of 10 mm, 20 mm, 30 mm, 40 mm, and 50 mm, respectively. Each simulation scheme included seven rock-size working conditions, and the rock sizes were 100 mm, 200 mm, 400 mm, 600 mm, 800 mm, 1000 mm, and 1200 mm. Numerical simulation of uniaxial compression was carried out for each working condition. The numerical simulation schemes are given in Table 1.

**Table 1:** Summary of simulation scheme and working conditions.

Simulation scheme	Parallel-joint spacing *s* [mm]	Rock size *l* [mm]						
		100	200	400	600	800	1000	1200
**1**	**10**	10×100	10×200	10×400	10×600	10×800	10×1000	10×1200
**2**	**20**	20×100	20×200	20×400	20×600	20×800	20×1000	20×1200
**3**	**30**	30×100	30×200	30×400	30×600	30×800	30×1000	30×1200
**4**	**40**	40×100	40×200	40×400	40×600	40×800	40×1000	40×1200
**5**	**50**	50×100	50×200	50×400	50×600	50×800	50×1000	50×1200

**Fig 2:**
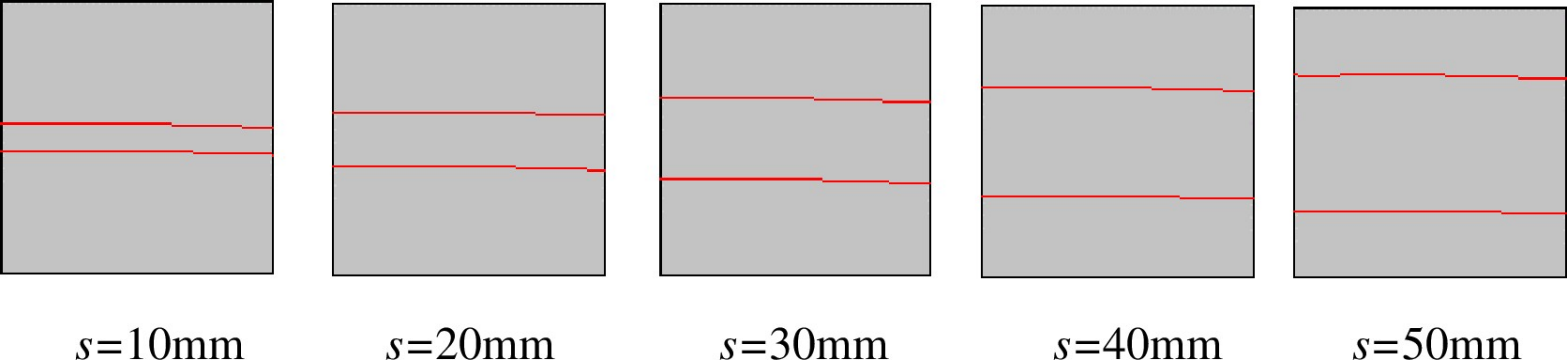
Simulation model of different parallel-joint spacings when the rock size *l* is 100 mm × 100 mm.

**Fig 3:**
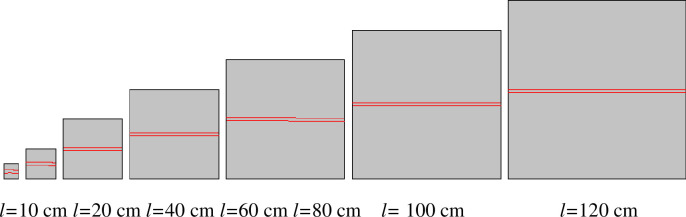
Simulation model of different rock sizes when the parallel-joint spacing *s* is 10 mm.

The simulations involved 35 working conditions and simulation models. However, to limit the article length, reported here are a series of simulation models with a rock size of 100 mm × 100 mm (Fig 2) and a series of simulation models with a PJS of 10 mm (Fig 3) as examples. Through these two examples, the process of establishing the simulation model when the PJS changes and the process of establishing the simulation model when the size of the rock changes are introduced.

### Boundary conditions and rock joint parameters

#### Boundary conditions and loading methods

**Fig 4:**
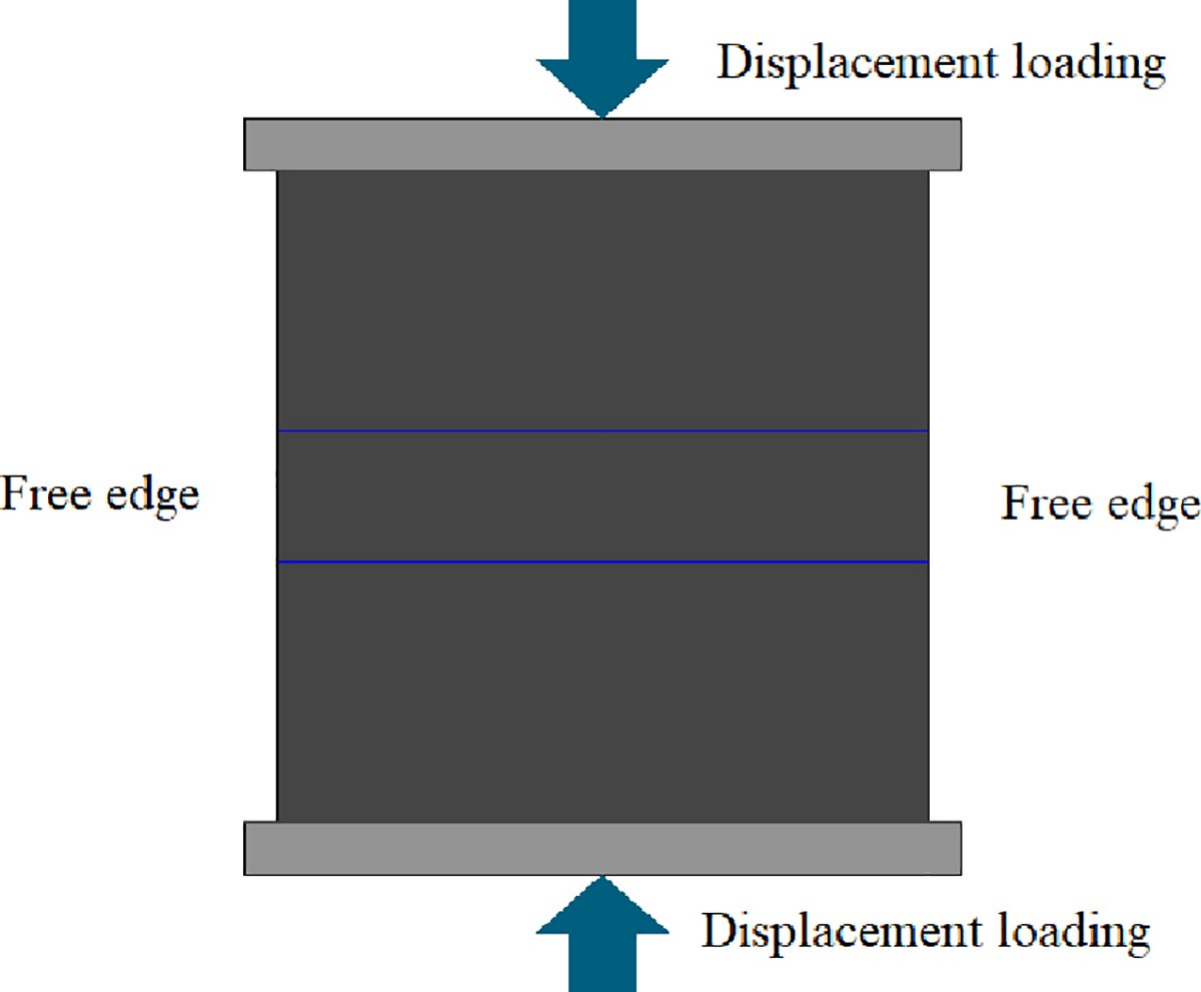
Uniaxial compression simulation loading method and boundary control model diagram.

Uniaxial compression deformation theory and the plane stress model were used in the simulation. The constraint conditions were that the load was borne not by the two sides of the model but by its upper and lower surfaces. The displacement loading method was adopted in the numerical simulation. The displacement loading on both sides of the model was zero, and that on its upper and lower surfaces was 0.01 mm. The judgment criterion used in the simulation was the Mohr–Coulomb criterion. The loading method and boundary control are shown in Fig 4.

#### Rock mechanical parameters and joint parameters

In this study, the rock mechanical parameters were obtained through a geological survey of the slope rock mass of Zhang’ao Mine, which is located in Zhang’ao Village, Shengzhou City, Zhejiang Province, China. First, the rock resilience measurement, structural surface measurement, and rock mass classification of the slope rock were carried out to determine the UCS and rock mass grade. Then, the rock mechanical parameters (e.g., elastic modulus, Poisson’s ratio, friction angle) were evaluated based on the Hoek–Brown criterion.

The elastic modulus of the rock was 4874 MPa, its Poisson’s ratio was 0.25, its compressive strength was 101.34 MPa, its cohesion was 1.2 MPa, its friction angle was 48.32°, its density was 2600 g/cm^3^, the normal stiffness of the PJ was 50 GPa/m, its tangential stiffness was 25 GPa/m, the friction angle was 40°, and the joint roughness coefficient (JRC) value was 2. The mechanical parameters of the rock are given in Table 2, and those of the joints as quoted from the literature [26] are given in Table 3.

**Table 2:** Mechanical parameters of rock.

Material	Elastic modulus [MPa]	Uniaxial compressive strength [MPa]	Poisson’s ratio	Friction angle [°]
Rock	4874	101.34	0.25	48.32

**Table 3:** Mechanical parameters of joints.

Material	Normal stiffness [GPa/m]	Tangential stiffness [GPa/m]	Friction angle [°]	JRC
Parallel joints	50	25	40	2

### Numerical simulation results and analysis

#### Analysis of stress–strain curves of parallel-jointed rock with different sizes

**Fig 5:**
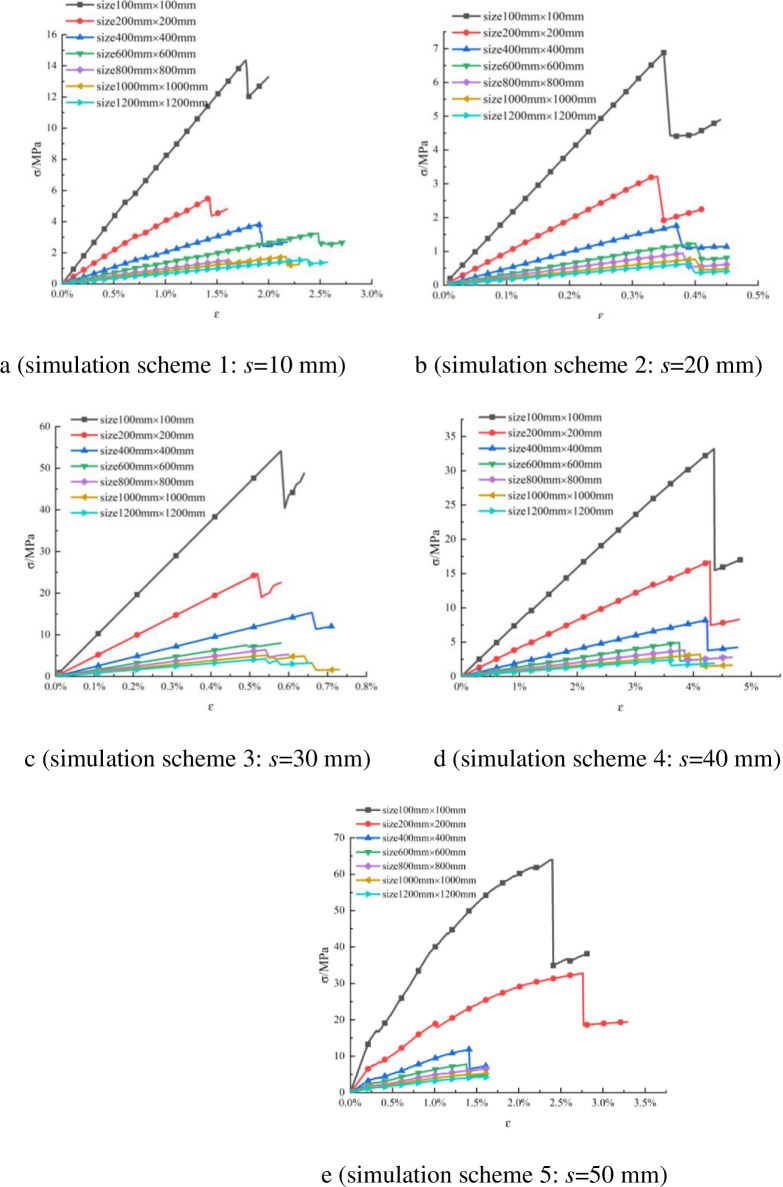
Stress–strain curves of different sizes of rock in uniaxial compression simulations.

For each simulation scheme, the numerical simulation results for each working condition were output, and the stress–strain curve for each working condition was drawn. Then the stress–strain curves for all working conditions in each simulation scheme were drawn in the same coordinate system, and a summary of the stress–strain curves of different rock sizes for each simulation scheme was obtained, as shown in Fig 5.

According to the stress–strain curve for each working condition in the simulation scheme, the UCS of the rock in each working condition was explored. The UCS under all working conditions is summarized in Table 4.

**Table 4:** Summary of uniaxial compressive strength under all working conditions.

Simulation scheme	Parallel-joint spacing [mm]	Uniaxial compressive strength of rock under different sizes [MPa]						
		100 mm	200 mm	400 mm	600 mm	800 mm	1000 mm	1200 mm
**1**	**10**	14.36	5.55	3.83	3.26	1.52	1.76	1.57
**2**	**20**	6.88	3.22	1.76	1.24	0.95	0.77	0.63
**3**	**30**	54.13	24.66	15.33	7.5	6.41	4.91	4.25
**4**	**40**	33.2	16.8	8.23	4.95	3.84	3.21	2.36
**5**	**50**	64.02	32.84	11.91	7.83	7.97	5.75	4.89

Because the stress–strain curves are similar, the curve for the rock size of 100 mm in Fig 5A is selected as an example to analyze the law of the stress–strain curve. When the stress is small, the stress–strain curve is approximately a straight line. When the stress increases to 14.36 MPa, the curve drops suddenly to 12.1 MPa. This shows that the rock exhibits obvious elastic and brittle characteristics, i.e., the stress increases linearly with the strain, then the strain continues to increase and the stress reaches its peak, at which point the strain energy inside the rock is released, causing the stress to drop rapidly. At the same time, the rock broke immediately after reaching the peak strength, which was sudden and accidental; this is also a characteristic of brittle materials, indicating that the rock has poor ductility.

Analyzing the influence of rock size on the UCS, Fig 5A and Table 4 show that the peak strength of the rock decreases from 14.36 MPa to 1.57 MPa as the size of the rock increases when the PJS is 10 mm. The law that the strength of the rock decreases with the increase of its size is obtained. In the same way, the same law is obtained when the PJS is 20 mm, 30 mm, 40 mm, and 50 mm, which shows that the size of the rock has an important influence on its UCS, i.e., the strength of the rock has an SE.

Analyzing the influence of PJS on the UCS of rock, Fig 5 and Table 4 show that the peak strength increases from 14.36 MPa to 64.02 MPa with the increase of the PJS when the rock size is 100 mm. The law that the rock strength increases with the increase of the PJS is obtained. In the same way, the same law is obtained when the rock size is 200 mm, 400 mm, 600 mm, 800 mm, 1000 mm, and 1200 mm, which shows that the PJS also affects the UCS of the rock.

In short, when the PJS is the same, the larger the size of the rock, the smaller its UCS. When the size of the rock is the same, the UCS of the rock increases with the increase of the PJS.

### Fitting method for relationship between uniaxial compressive strength and parallel-jointed rock size

**Fig 6:**
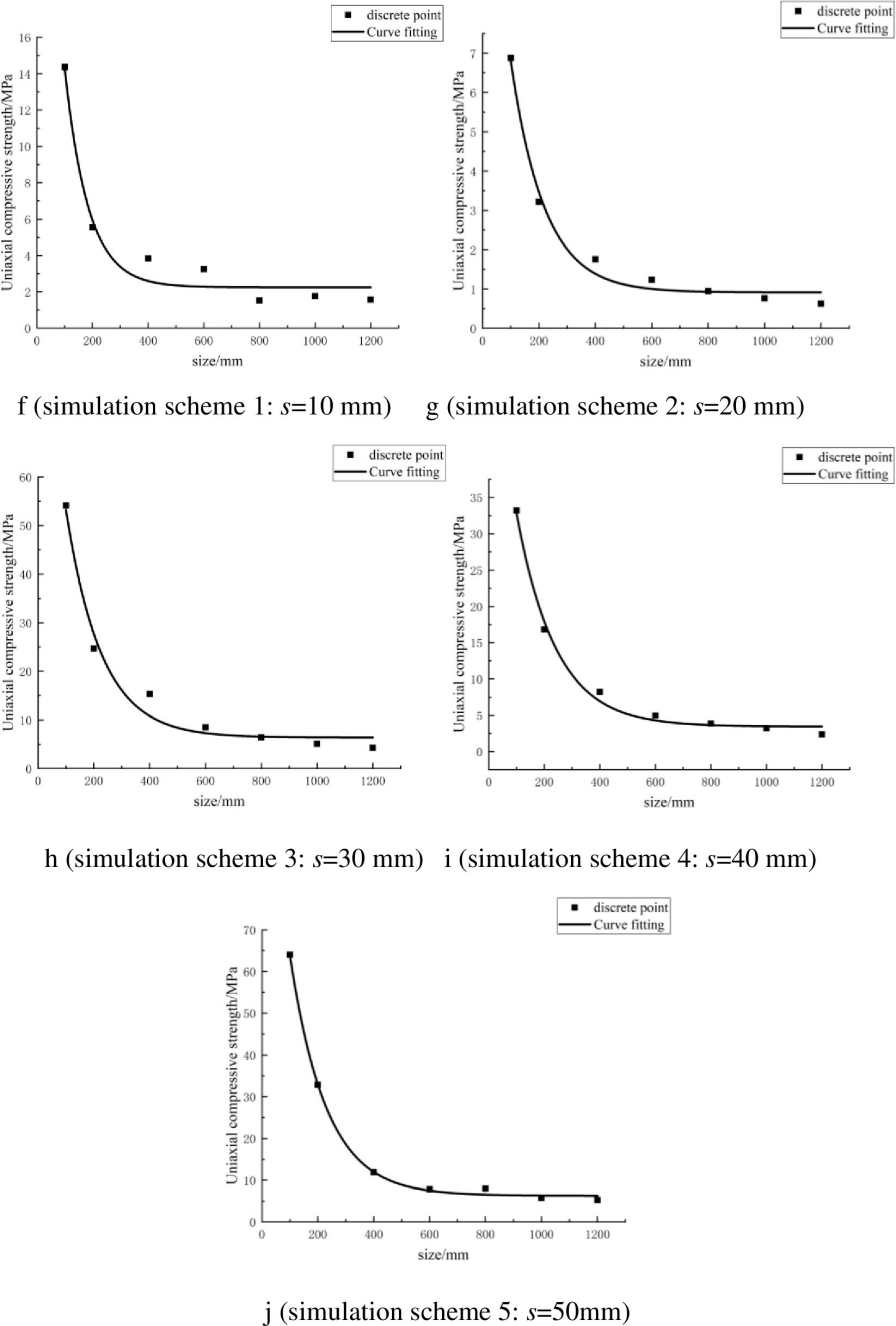
Fitting curve of uniaxial compressive strength and size of rock with parallel joints.

The statistics in Table 4 show that as the size of the rock increases, its UCS gradually decreases. A scatter diagram of the UCS and rock size in each numerical simulation scheme was drawn. Then, according to the scatter diagram, the fitting curve of the UCS and rock size was drawn, as shown in Fig 6.

The fitting curves in Fig 6 show that the UCS of the rock gradually decreases as the size of the rock increases under the same PJS. Finally, it tends to a stable value. Rocks with different PJS have the same changing law. From the fitting curves in Fig 6F–6J, the following relationships between the UCS and the size of the PJed rock were obtained:

$$\sigma (l)=2.25+39.18e^{-0.0118l}$$ (9)

when the PJS was 10 mm,

$$\sigma (l)=0.917+13.65e^{-0.0084l}$$ (10)

when the PJS was 20 mm,

$$\sigma (l)=6.391+102.82e^{-0.0079l}$$ (11)

when the PJS was 30 mm,

$$\sigma (l)=3.444+59.64e^{-0.0071l}$$ (12)

when the PJS was 40 mm, and

$$\sigma (l)=6.273+124.75e^{-0.0077l}$$ (13)

when the PJS was 50 mm, where *σ*(*l*) [MPa] is the UCS of the rock and *l* [mm] is the size of the rock.

### Relationship between uniaxial compressive strength and parallel-jointed rock size

#### Mathematical model (1)

Eqs 9–13 are the relationships between UCS and rock size with different PJS. The function types of these formulas were analyzed, and the following mathematical model for rock UCS versus the size of the rock with PJs is proposed:

$$\sigma (l)=a+be^{\left(-cl\right)}$$ (14)

where *σ*(*l*) [MPa] is the UCS of the rock when the size of the PJed rock is *l* [mm], and *a*, *b*, and *c* are undetermined parameters.

Eq 14 is the mathematical relationship between the UCS and the size of the rock with PJs. This formula contains undetermined parameters *a*, *b*, and *c*. When *a*, *b*, and *c* are determined, the relationship between the UCS and the size of the rock containing PJs can be obtained.

#### Method for evaluating parameters

**Fig 7:**
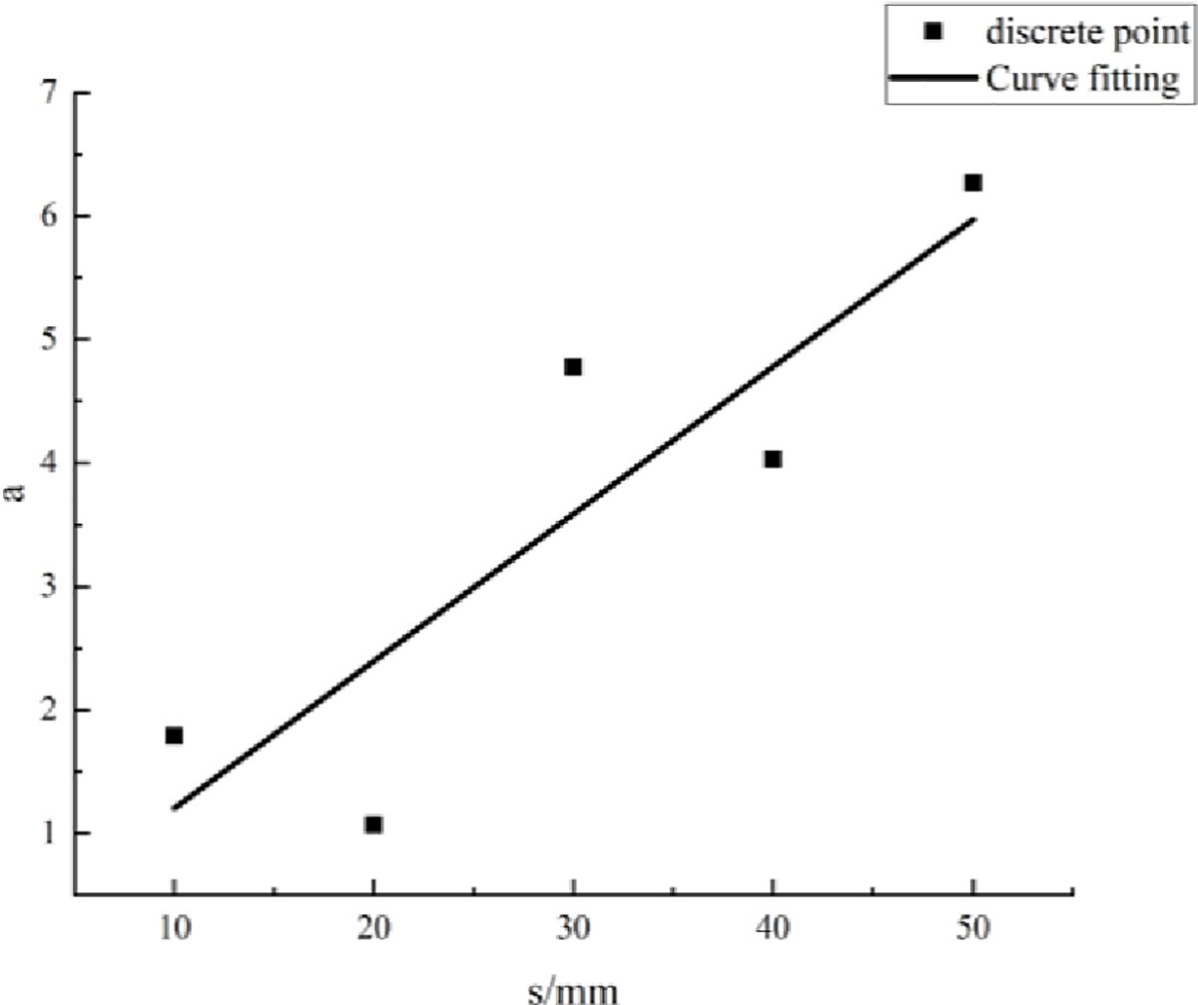
Fitting curve of parameter *a* and parallel-joint spacing.

**Fig 8:**
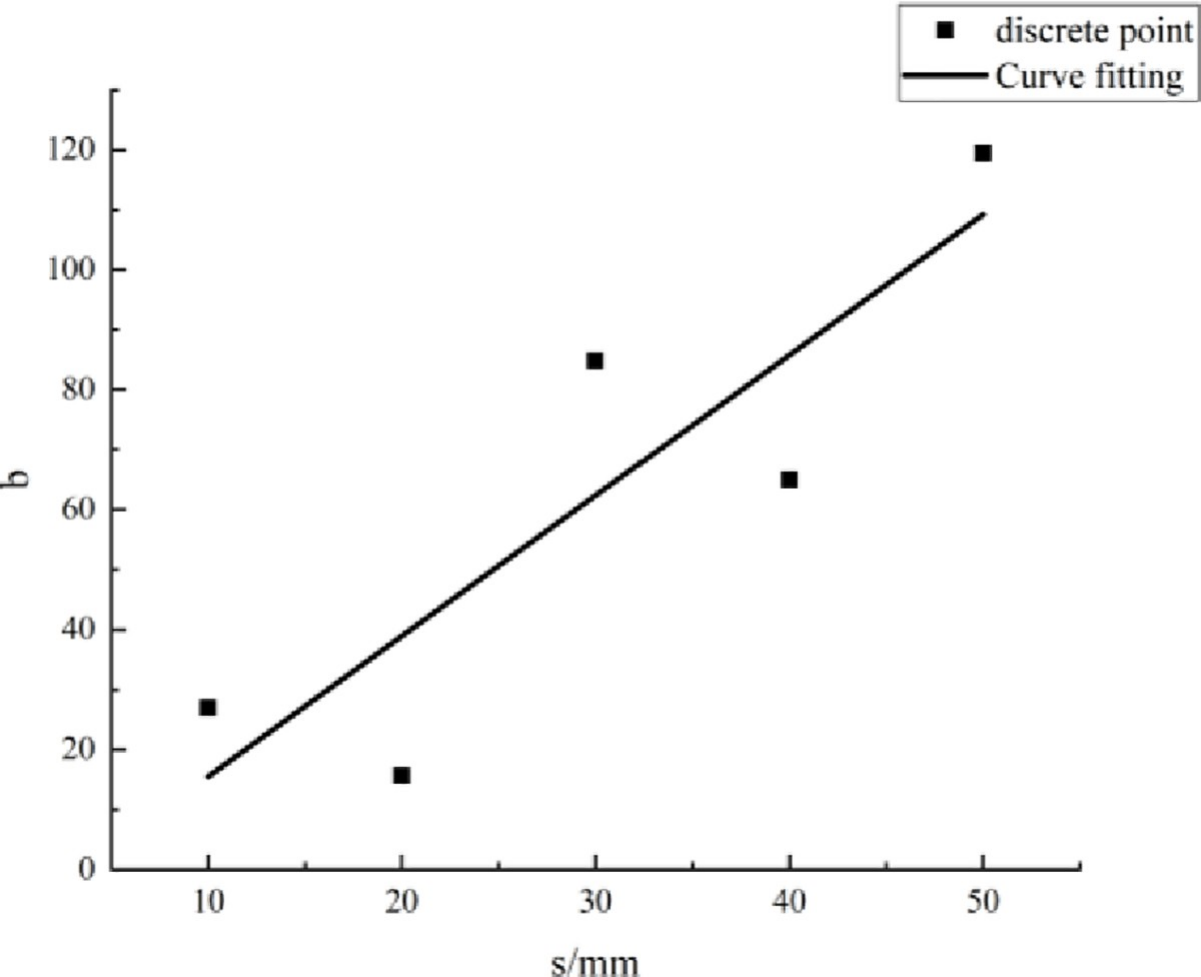
Fitting curve of parameter *b* and parallel-joint spacing.

**Fig 9:**
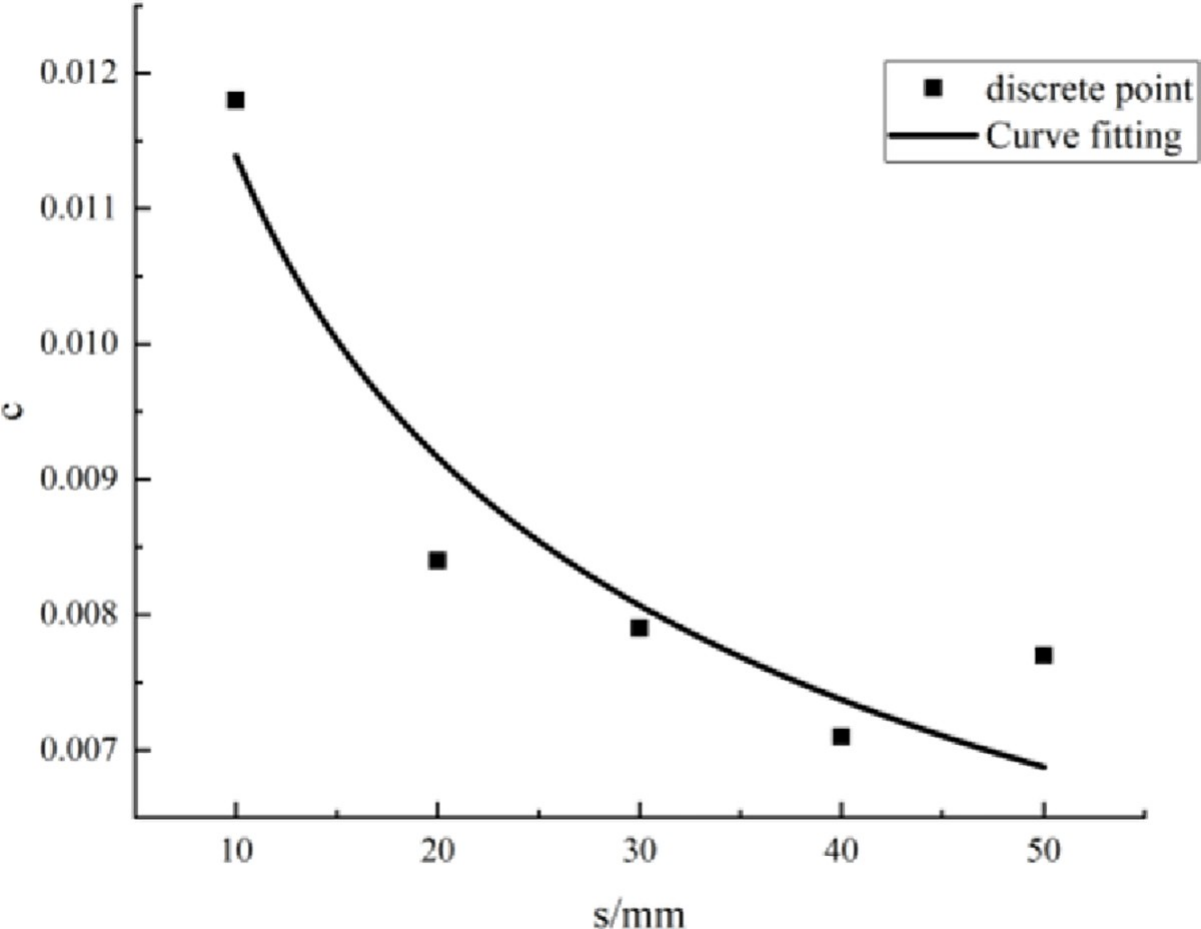
Fitting curve of parameter *c* and parallel-joint spacing.

Eqs 9–13 show that the parameter values are related to the PJS. The parameters under each PJS were calculated and are summarized in Table 5. According to the data therein, a scatter diagram of the parameters and the PJS was drawn, with the PJS taken as the abscissa and the parameter value taken as the ordinate. Then, according to the scatter diagram, the relationship between the parameters *a*, *b*, and *c* and the PJS was fitted, as shown in Figs 7–9.

**Table 5:** Values of parameters *a*, *b*, and *c*.

Parameter	Parallel-joint spacing *s* [mm]				
	10	20	30	40	50
***a***	1.79	1.07	4.775	4.03	6.27
***b***	26.97	15.6	84.77	65	119.4
***c***	0.0118	0.0084	0.0079	0.0071	0.0077

According to the fitting curves in Figs 7–9, the relationships between the parameters *a*, *b*, and *c* and the PJS *s* are as follows:

a=0.119s+0.011 (15)

b=2.343s−7.93 (16)

$$c=0.024s^{-0.314}$$ (17)

#### Mathematical model (2)

The parameters *a*, *b*, and *c* in Eq 14 are unknown and are established using Eqs 15–17. Therefore, the specific form of the relationship between the UCS and the size of the rock with PJs is

$$\sigma (l)=0.119s+0.011+(2.343s-7.93)e^{-0.024ls^{-0.314}}$$ (18)

where *σ*(*l*) [MPa] is the UCS when the size of the rock with PJs is *l* [mm], and *s* [mm] is the PJS.

Eq 18 provides a method for quantitatively analyzing the UCS and size of the rocks with PJs, one that can be extended to evaluate the compressive strength of similar rocks with PJs. This formula makes it easier and faster to evaluate the compressive strength of rocks with PJs.

### Relationship between compression characteristic size and parallel-joint spacing

In rock mechanics, the characteristic size is usually used to characterize the SE of the rock. The compressive strength of rock has an SE, therefore the SE of the compressive strength can be characterized by the characteristic size of the compressive strength. It is of great significance to analyze the influence of PJS on the characteristic size of rock compressive strength.

In statistics, events with a probability of less than 5% are usually referred to as “impossible events.” The characteristic size of compressive strength can be determined accurately by using a mathematical significance test. The quantitative calculation of characteristic size is described in detail in the literature [12] based on physical and mechanical experiments and the statistical distribution theory of meso-parameters. According to the research ideas of the literature [12], the absolute value of the slope of the curve was obtained by deriving the two sides of Eq 14:

$$\left|k|=|bce^{\left(-cl\right)}\right|$$ (19)

|k|≤α (20)

$$l\geq \frac{\ln(bc)-\ln\alpha }{c}$$ (21)

where *α* is the acceptable absolute value of the slope, which can be a very small value. If the approval curve is close to the level and meets the engineering requirements, then Eq 21 is considered the formula for evaluating the characteristic size.

Herein, the rock size is obtained when the significance level is 5% and is taken as the characteristic size of rock compressive strength. When the PJS was 10 mm, 20 mm, 30 mm, 40 mm, and 50 mm, the characteristic sizes of the rock compressive strength were calculated as summarized in Table 6.

**Table 6:** Relationship between characteristic size and parallel-joint spacing.

Parallel-joint spacing [mm]	10	20	30	40	50
**Characteristic size [mm]**	578.76	647.04	880.12	949.52	981.91

**Fig 10:**
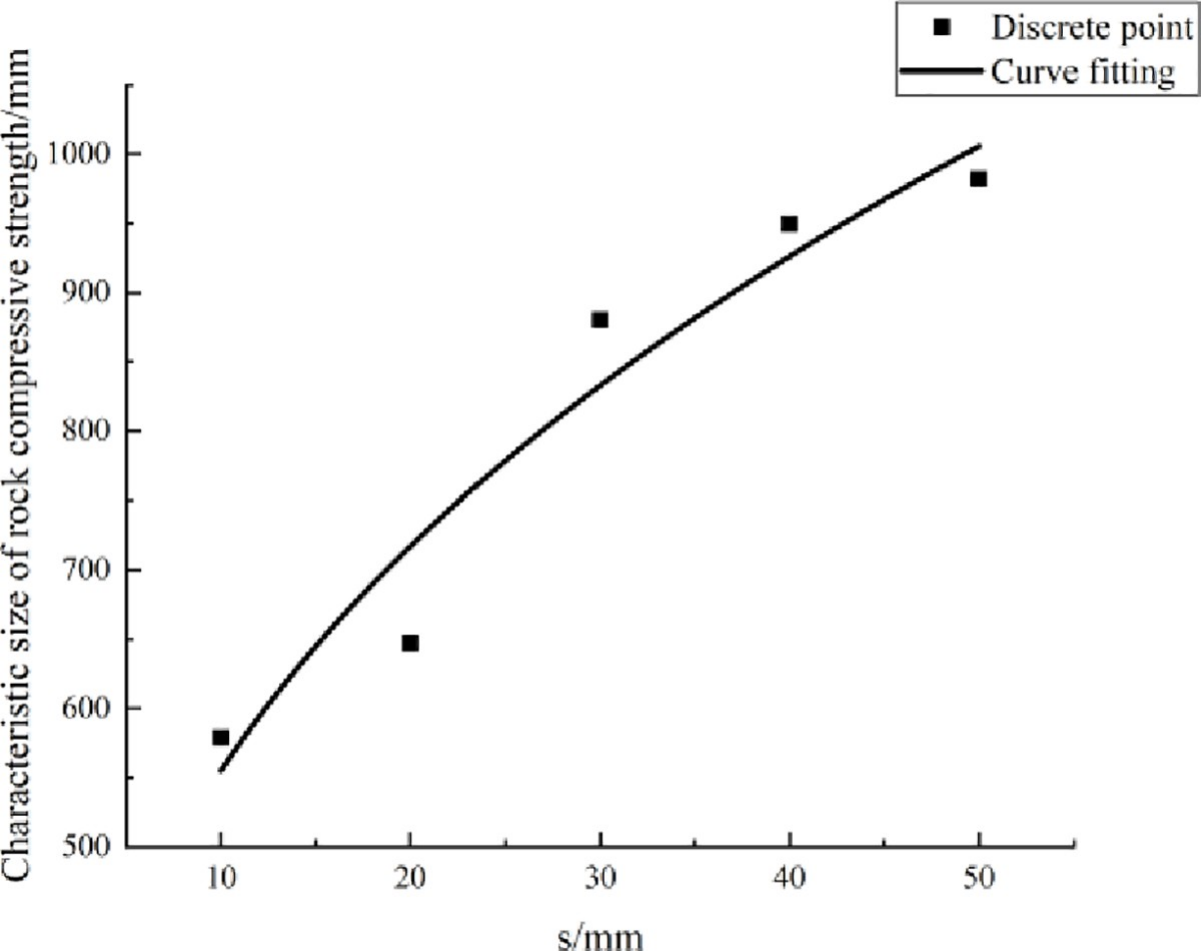
Fitting curve of uniaxial compression characteristic size and parallel-joint spacing.

The data in Table 6 show that as the PJS gradually increases, the characteristic size of compressive strength gradually increases. A scatter diagram of the characteristic size of compressive strength and PJS was drawn, with the PJS taken as the abscissa and the characteristic size taken as the ordinate. Then, according to the scatter diagram, the relationship between the characteristic size and the PJS was fitted, as shown in Fig 10.

Fig 10 shows that the characteristic size of compressive strength shows an increasing trend with the increase of the PJS, and the curve conforms to the relationship of a power function. The specific form of the relationship between the characteristic size and PJS is

$$D=237.299s^{0.369}$$ (22)

where *D* [MPa] is the uniaxial compressive characteristic size and *s* [mm] is the PJS.

Eq 22 provides a way to analyze quantitatively the relationship between the characteristic size of compressive strength and the PJS. It can be extended to evaluate the characteristic size of the compressive strength of similar rocks with PJs. In engineering, if the PJS of the rock can be measured, then the characteristic size of compressive strength can be calculated accurately by Eq 21. Obtaining this equation is of great significance for studying the SE of rock compressive strength.

## Discussion

The size of the rock with PJs has an influence on the UCS, and the PJS has an influence on the characteristic size of the compressive strength, but their relationships have not yet been obtained. In this paper, a mathematical model of compressive strength and the size of rock with PJs was established, as was a mathematical model of the characteristic size of compressive strength and PJS. Acquiring these two mathematical models has important scientific significance. For engineering, once the on-site PJS and the size of the rock containing PJs are obtained, the compressive strength and its characteristic size can be evaluated quickly, which has important engineering guidance significance.

This paper provides a method for solving the relationship between rock compressive strength and the size of rock with PJs, as well as a method for solving the relationship between the characteristic size of compressive strength and PJS. At the same time, this paper also provides a method for evaluating the parameters in the mathematical model. For a particular rock with PJs, using the method in the paper, mathematical models suitable for the rock can be obtained.

However, this paper still has shortcomings. This study was carried out by means of numerical simulation, simplifying the simulation conditions. The study simulated the SE of the compressive strength of two-dimensional rock and did not carry out a three-dimensional simulation. When the numerical model was established, only two PJs in the rock were considered, and other factors in the rock, such as small cracks and water content, were ignored. Therefore, the factors considered are not yet complete, and there are still areas for improvement. When the research results are extended to the project site, they are only applicable to specific rocks with PJs.

## Conclusions

This study investigated the influence of the size of PJed rock on the UCS and the influence of the PJS on the characteristic size of the compressive strength. The stress–strain curves of the uniaxial compression of PJed rock with different sizes were investigated numerically. From these simulations, the following conclusions can be drawn. The relationship between the UCS and the size of the PJed rock is

$$\sigma (l)=a+be^{\left(-cl\right)}$$

and in particular our simulations obtained

$$\sigma (l)=0.119s+0.011+(2.343s-7.93)e^{-0.024ls^{-0.314}}.$$

The characteristic size of the rock uniaxial compression specimen is found to be related to the PJS, and our simulation gives the particular form

$$D=237.299s^{0.369}.$$
